# Healthcare seeking behaviors for common childhood illnesses among caregivers of children under 5 years of age in Palestine: a cross-sectional study

**DOI:** 10.1186/s12889-026-26632-w

**Published:** 2026-02-18

**Authors:** Mays Othman, Dima Abu-Hanieh, Roaa Turkman, Rasha Khayyat, Reem Sawafta

**Affiliations:** 1 Faculty of medicine and allied medical sciences, An-Najah National University, Nablus, Palestine; 2 Department of Biomedical Sciences and Basic Clinical Skills, Faculty of Medicine and Allied Medical Sciences, An-Najah National University, Nablus, Palestine; 3 Medical Sciences Research Unit, Medical and Health Sciences Research Center, Scientific Centers, An-Najah National University, Nablus, Palestine; 4 Department of Medicine, Faculty of Medicine and Allied Medical Sciences, An-Najah National University, Nablus, Palestine

**Keywords:** Health care, Public health, Caregivers, Palestine, Children under the age of 5, Health care seeking

## Abstract

**Background:**

Caregivers’ healthcare-seeking behaviors (HSB) are critical in reducing child mortality and morbidity, particularly in developing countries. Children comprise a large proportion of the Palestinian population, yet under-five mortality remains a concern due to many preventable diseases. This study aimed to assess HSB and to determine the prevalence and predictors of appropriate HSB among caregivers of children under 5 years of age during common childhood illnesses in the West Bank in 2024.

**Methods:**

A cross-sectional study was conducted in the West Bank from June to August 2024, using a validated questionnaire developed by Webair et al. administered via face-to-face interview with a convenience sample of parents attending primary healthcare centers in four governorates. Data analysis included descriptive statistics, bivariate analysis by chi-square test, and multivariable logistic regression to identify independent predictors.

**Results:**

A total of 427 caregivers were interviewed; overall, 55.5% (95% CI: 50.8%–60.2%) demonstrated appropriate HSB. However, many initially relied on traditional or self-treatment methods, leading to delays in seeking professional care. Logistic regression analysis identified caregivers’ perceptions of illness as severe (OR = 2.43, 95% CI: 1.60–3.72). and the presence of specific symptoms, particularly respiratory issues (OR = 1.87, 95% CI: 1.11–3.16) and fever (OR = 1.61, 95% CI: 1.02–2.54), as significant predictors of appropriate HSB. Along with the place of residence (OR = 0.57, 95% CI: 0.36–0.90), caregivers in cities are less likely than those in villages or camps to engage in appropriate HSB.

**Conclusion:**

We conclude that many caregivers delay seeking professional care, often relying on traditional or self-treatment, and that timely health-seeking is influenced by caregivers’ perceptions of illness severity, specific symptoms, and place of residence. These findings highlight the need for targeted interventions to support appropriate health-seeking behavior.

## Background

Caregiver’s healthcare-seeking behaviors (HSB) refer to a caregiver’s response to illness signs and symptoms to reduce the severity, complications, or even death after a child’s illness has been identified [1]. Appropriate HSB and access to healthcare services are important in preventing many child deaths, particularly in developing countries [2]. In Palestine, children account for 43% of the total population (41% in the West Bank and 47% in the Gaza Strip), with approximately 30% of them being under 5 years of age in the West Bank [3]. Globally, children younger than 5 years are vulnerable to infectious diseases such as pneumonia, diarrhea, and malaria, and these diseases are considered the leading causes of death among them [4]. The Under-five Mortality (U5M) rate in the West Bank in 2023 was 11.1 deaths per 1000 live births, according to the Palestinian Ministry of Health’s Annual Health Report [5]. The World Health Organization (WHO) has stated that more than half of all child deaths could be easily prevented or treated if children were given access to healthcare [2]. The entire world is currently working hard to reduce child mortality and improve children’s quality of life.

Health service provision in Palestine is delivered through four main sectors: the governmental services (the Palestinian Ministry of Health and Military Medical Services), the United Nations Relief and Works Agency (UNRWA), non-governmental organizations, and the private sector. Across these sectors, citizens have access to primary, secondary, and tertiary levels of care. The Palestinian health system places a high value on keeping Palestinian children healthy and safe from disease and death [6]. According to the Palestinian Ministry of Health’s Annual Health Report, since 2016, children up to the age of 6 years have been receiving free healthcare services at its centers, an increase from the previous limit of three years. In 2023, there were 521,863 visits by children under three years to primary healthcare centers in the West Bank [5]. Based on data from the Palestinian Central Bureau of Statistics, the count of children under 5 years of age was 413,194; assuming an even age distribution, the under-3 population is estimated at 247,916, yielding an average of about 2.1 visits per child [7], although health care facilities are apparently available, physical accessibility to address timely child healthcare is frequently challenged by prolonged fragmentation under military occupation, such as checkpoints, the Separation Barrier, and a lack of transfer permissions, reinforcing delays and disruptions in care. These structural constraints are compounded by economic hardship, uneven service provision, and a fragmented health system, with the Ministry of Health as the main provider. The Ministry has faced frequent interruptions in service delivery and reduced actual working days due to strict regulations and financial constraints [8, 9]. Additionally, recent conflict has caused a substantial drop in monthly family incomes, further limiting caregivers’ ability to seek care. Moreover, the prevailing psychosocial burden -rooted in chronic exposure to conflict and trauma -can deter proactive care-seeking, amplify mental stress among caregivers, and influence decisions around when and how to seek healthcare for their children [10], Delays in seeking healthcare for Palestinian children undermine children’s fundamental right to timely and appropriate health care, and adversely affect health outcomes like that observed in other developing countries, such as disease progression and increased risk of mortality, particularly in cases of pneumonia and other critical illnesses [11–13].

Barriers to access to official medical care may lead families to rely on complementary and alternative medicine (CAM). In a previous study, 100% of parents reported having used traditional therapies at least once in their lifetimes [14]. The use of CAM carries potential risks, particularly because it is not always based on scientific evidence, and there are no formal policies to ensure its safety and efficacy.

In Palestine, parents, particularly mothers, are the primary caregivers for young children; they are usually responsible for daily childcare and health-related decision-making. Other caregivers, such as grandparents or extended family members, may also assume caregiving roles, especially in extended family settings or when mothers are unavailable, for example, after parent separation, or upon maternal employment. Previous evidence suggests that caregivers’ socio-demographic characteristics, including age, educational level, employment status, and relationship to the child, can influence healthcare-seeking behavior [15–17]. Similarly, child-related factors such as age and sex may affect caregivers’ perceptions of illness severity and decisions regarding when and where to seek care. Understanding these caregiver- and child-related characteristics is essential for identifying factors associated with appropriate healthcare-seeking behavior among children under five years of age [16–18].

Little is known about the extent of child caregivers’ HSB in Palestine. To the researcher’s knowledge, there was no data on caregivers’ decision-making, timing of care-seeking, and factors influencing appropriate versus delayed healthcare utilization. And here came the necessity of this study, which aimed to assess HSB and determine the prevalence of appropriate caregivers’ HSB in the West Bank in 2024 for common childhood illnesses and the factors related to these behaviors. Which could provide health policies with baseline data to improve health services and develop programs to educate caregivers about appropriate HSB, thereby improving childhood survival and quality of life while also contributing to broader global health goals, such as the Sustainable Development Goals (SDGs).

## Method

### Study design

A descriptive, analytical cross-sectional study design was used to address the research goal. The study took place in the West Bank of Palestine from June 2024 to August 2024, with a total sample size of 427 participants.

### Target population

The study included parents or guardians who were caring for children under 5 years of age. Eligible participants were caregivers of children from this age group who had experienced an acute illness (diarrhea, fever, cough, and/or difficulty breathing) within the preceding 14 days, a time frame chosen to minimize recall bias, and who were willing to participate and provided written informed consent. Caregivers who declined participation were those who didn’t meet the inclusion criteria or had limited ability to participate in or complete the interview.

### Sampling

The sample size was calculated using the Raosoft online sample size calculator, based on the estimated number of children under five years of age in the West Bank (408,109), as reported by the Palestinian Central Bureau of Statistics (PCBS) [7]. The calculation was intended to estimate the prevalence of appropriate health-seeking behavior with reasonable precision. Although the study was not specifically powered to detect associations between explanatory variables and the outcome, such associations were explored analytically. The calculation assumed a 50% response distribution, a 5% margin of error, and a 95% confidence level, resulting in a minimum required sample size of 384 participants. To account for potential non-response, the sample size was adjusted using the standard non-response adjustment method, applying a 10% non-response rate consistent with previous Palestinian population surveys, yielding a final target sample size of 427 participants.

The research was conducted in the Palestinian West Bank governorates of Nablus, Jenin, Tulkarm, and Ramallah Based on the investigators’ convenience and because these governorates were chosen because they are considered the most populated districts in the north and center of the West Bank, each of these Governorates has a total population of 431,600, 352,900, 205,900, and 370,000 people, respectively, making up approximately 42% of the Palestinian population in the West Bank [19]. Participants were recruited from each governorate in proportion to the population of the governorate, resulting in 135 participants from Nablus, 111 from Jenin, 62 from Tulkarm, and 116 from Ramallah.

Participants were recruited from 14 primary healthcare centers related to the Palestinian Ministry of Health located in cities, villages, and refugee camps across the selected governorates. Caregivers routinely attend these centers for childhood vaccination services, which are universally utilized regardless of socioeconomic background, ensuring broad population representation. A convenience sampling technique was used to recruit participants in each primary healthcare center during the days of data collection.

### Data collection and data collection tool

Data was collected using face-to-face interviews with caregivers in quiet, private rooms in the primary healthcare centers on vaccination days. All interviews were conducted directly by the researchers themselves, who received training before the data collection process to standardize the questioning style and approach and to avoid interviewer bias. To reduce social desirability bias, participants were reminded that their responses were confidential and solely for research purposes, encouraging honest reporting of health-seeking behavior. The interviews were conducted in Arabic and were based on a previously developed and validated questionnaire by Webair and Bin-Gouth titled “Questionnaire about Factors Affecting Health Seeking Behavior for Common Childhood Illnesses in Shehair City.“ [15]. It was created in Arabic to identify factors influencing HSB for childhood illnesses in Yemen. During the interview, researchers clarified the meaning of each questionnaire item and addressed any questions or concerns raised by the caregivers.

The questionnaire was divided into five sections and contained 16 questions in addition to the child’s and caregivers’ sociodemographic characteristics in the first section. Closed-ended questions with multiple answers, such as “Yes”, “No”, and “Sometimes”, as well as open questions.

The first section contained nine items that focused on caregivers and children’s sociodemographic characteristics; the second section contained two items that assessed disease characteristics; the third section evaluated caregivers’ HSB by determining actions taken for the complaint (first, second, and third if present); and the fourth section evaluated the Rationale of HSB. Finally, the fifth segment evaluated health service features.

Content validity was ensured through review by experts in pediatrics and public health, who assessed the relevance, clarity, and comprehensiveness of all items. Minor adjustments were made based on their feedback to improve clarity and applicability to the study population. A pilot study was conducted on 23 caregivers before the formal data collection process to ensure the instrument’s quality, coverage, and appropriateness for the intended measurement goal.

The questionnaire used for data collection is available upon request.

### Study variables

Our study included a dependent variable and several independent variables. The dependent variable in our study was appropriate HSB.

The independent variables included caregivers’ sociodemographic characteristics (age, marital status, educational status and residence), child characteristics (age, sex, birth order and number of siblings), illness characteristics (perceived severity of the illness, specific symptoms as fever, diarrhea, cough and difficulty breathing), and health service’s characteristics (accessibility, acceptability and affordability).

#### Operational definitions

To address the study aims, the variables were operationally defined to ensure consistent understanding and interpretation.

- Appropriate HSB: was defined as recognizing the need to seek medical care in the first three days of a child’s complaint from a modern healer (appropriate health facilities, primary health centers, hospitals, and private clinics), regardless of whether it is the first, second, or later action taken by the caregiver [15, 20].
- Inappropriate HSB: referred to consulting a pharmacist for medical care, self-treatment at home and using home remedies, visiting traditional healers, or not seeking any care during the illness of children under 5 years of age, or delayed seeking medical care [15, 20, 21].
- Delayed seeking of medical care: specifically described as seeking medical help more than three days after the onset of the complaint [15].
- The rationale for seeking medical care was defined as the cause of seeking medical care.
- Time of seeking medical care: referred to the duration during which the complaint was present before seeking medical care.
- Perceived severity of the illness: represented a subjective evaluation of illness by caregivers based on discomfort present in the child, and it was categorized as severe or not.

Finally, common childhood illnesses covered in our study included infectious diseases, respiratory diseases, gastrointestinal diseases, and other diseases characterized by fever, diarrhea, cough, or difficulty breathing.

### Data analysis

The researchers screened the caregivers’ responses for completeness and consistency; incomplete responses were excluded, and the data were coded, checked, and processed with the Statistical Package for the Social Sciences (SPSS) version 22. Descriptive statistics were used to summarize variables; continuous data were presented by means/median, standard deviations (SD), and categorical data were expressed as frequencies and proportions. For further analyses, continuous variables were categorized, and categories of selected categorical variables were grouped based on scientific rationale and contextual similarity. To determine the association between HSB and other variables, statistical analysis with two sequential steps was conducted. First, bivariate analyses by Chi-square tests were done for all factors considered as predictors of HSB according to the literature, including socio-demographic characteristics of the caregivers and children, illness- and health services-related characteristics, and caregivers’ attitudes about traditional and modern medicine. Variables significantly associated during the chi-square test at p-value < 0.05 were considered for the multivariate logistic regression analysis, to ensure the stability of the regression model and to avoid over-fitting. Then the multivariate logistic regression analysis was applied, adjusted odds ratios were calculated in the presence of more than one explanatory variable in the model to control confounding, and the variables that were significant with a p-value < 0.05 were identified as associated factors of appropriate HSB. The results of the analysis were presented in texts, tables, and charts accordingly.

### Public involvement

The public, represented by the caregivers, was involved in the research planning process from the beginning. The initial idea of the research was inspired by frequently observed concerns and inquiries expressed by caregivers about the appropriate management of their children’s illnesses. Some caregivers were consulted before the study, and they suggested their preference for face-to-face interviews, which was taken into account in the data collection method. During the data collection process, they were actively involved in the time management of the interview. We were flexible enough to ask some questions before the child received the vaccine and others after, depending on the caregiver’s preferences. In addition, caregivers will be involved in planning the dissemination of results, including the distribution of brochures at primary healthcare centers, the sharing of awareness videos on social media, caregiver meetings, and educational campaigns.

### Ethical consideration

Before initiating the study, ethical approval was obtained from the Institutional Review Board (IRB Ref:105) of An-Najah National University and the Palestinian Ministry of Health (MOH) in the West Bank, covering all aspects of the research procedures. Additionally, permission to use the “Questionnaire about Factors Affecting Health Seeking Behavior for Common Childhood Illnesses in Shehair City” was obtained from the corresponding author. Furthermore, only participants who agreed to participate in the study (via a written consent form) after being informed of the nature and objectives of the study were considered. Moreover, researchers confirmed that the collected data was used for public health research only, while the information was confidential and was not used for any purpose other than the study.

## Results

### Socio-demographic characteristics of caregivers and children under 5 years of age

Of the 469 caregivers who met the inclusion criteria, 427 completed the interview, yielding a response rate of 91.0%. Among respondents, 94.6% were mothers, with a mean age of 29.6 years (SD ± 5.7), and 26.4% were employed. The children’s mean age was 22.7 months (SD ± 16.2), ranging from 14 days to 59 months. The number of siblings per child ranged from 0 to 7, with a median of 1. Table 1 provides the summary statistics for the socio-demographic characteristics of the study population.

**Table 1:** Socio-demographic characteristics of caregivers and their children under 5 years of age (*n* = 427)

Socio-demographic characteristics		Frequency (*n* %)
Caregivers characteristics		
Relation to the child	Mother	404 (94.6%)
	Father	20 (4.7%)
	Other	3 (0.7%)
Caregiver age (years)	≤ 29	220 (51.5%)
	> 29	207 (48.5%)
Marital status	Married	426 (99.8%)
	Single	1 (0.2%)
Occupation	Employed in the medical field	16 (3.7%)
	Employed in other fields	97 (22.7%)
	Unemployed	314 (73.5%)
Education	No formal education	1 (0.2%)
	Primary-education	28 (6.6%)
	Secondary-education	105 (24.6%)
	University education	293 (68.6%)
Place of residence	City	298 (69.8%)
	Village	120 (28.1%)
	Palestinian refugee camp	9 (2.1%)
Children characteristics		
Child age (months)	≤ 12	153 (35.8%)
	> 12	274 (64.2%)
Child sex	Male	234 (54.8%)
	Female	193 (45.2%)
Birth order	The first	159 (37.2%)
	The second	101 (23.7%)
	The third	80 (18.7%)
	The fourth or higher	87 (20.4%)
Number of siblings	≤ 2	311 (72.8%)
	> 2	116 (27.2%)

### Reported childhood illness

Fever was the most commonly reported symptom by 292 (68.4%) of study participants, followed by cough 189 (44.3%), diarrhea 150 (35.1%), and difficulty breathing 92(21.5%). Approximately 218 (51.1%) of the respondents perceived their children’s illnesses as severe.

### Healthcare-seeking behaviors

Of the 427 caregivers enrolled, 237 (55.5%) demonstrated appropriate healthcare-seeking behavior (95% CI: 50.8%–60.2%). In contrast, 166 (39.8%) did not seek medical care, and 24 (5.6%) sought care more than three days after the onset of illness; both were classified as inappropriate healthcare-seeking behavior. Among the 237 children who received medical care within the timeframe defined as appropriate healthcare-seeking behavior, 119 (50.2%) sought care on the second day after the perceived onset of illness, 94 (39.8%) on the first day, and 24 (10.1%) on the third day.

**Fig. 1:**
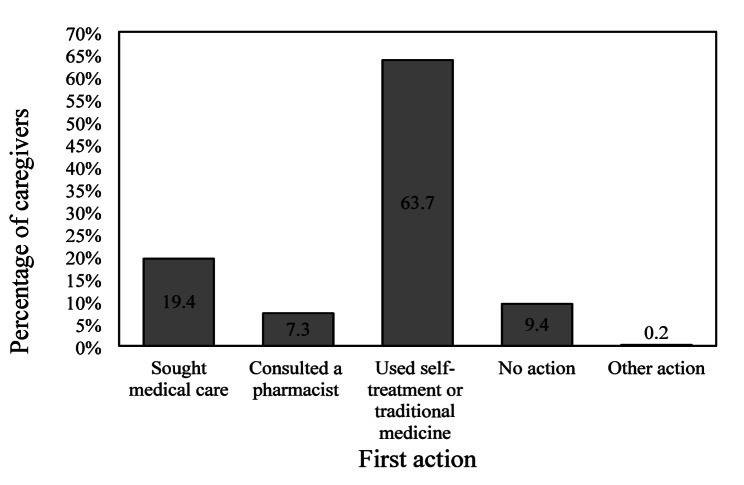
First action taken by caregivers of children under 5 years of age during their illness (*n* = 427)

Although 55.5% of caregivers ultimately demonstrated appropriate healthcare-seeking behavior, defined as seeking medical consultation within three days of illness onset, this was rarely the first action taken. The most common initial response to childhood illness was self-treatment or the use of traditional remedies (Fig. 1). Seeking medical care was the first action for only 83 caregivers (19.4%).

When caregivers were asked about who decided whether and when to seek medical care, 325 (76.1%) respondents reported that the mother alone made the decision. Another 78 (18.3%) stated that both parents made the decision. Nevertheless, the father made the decision alone in 18 (4.2%) of the cases, while other caregivers or relatives did so in 6 (1.4%) of the cases.

### Rationale of healthcare-seeking

Among caregivers who sought medical care (either within the appropriate time frame or delayed), the most frequently reported reason was that the illness had not resolved or had worsened, cited by 212 (81.2%) caregivers. This was followed by previous positive experiences with medical care (68; 26.2%) and advice from family or friends (23; 8.8%), as shown in Table 2.

**Table 2:** Causes of seeking medical care (*n* = 261)

Causes of seeking medical care	Frequency (*n* %)
Illness not resolved or worsened	212 (81.2%)
Good experience with medical care	68 (26.2%)
Family and friends’ advice	23 (8.8%)
Bad experience with traditional medicine	3 (1.2%)
Geographical access to health facilities	15 (5.8%)
Others	4 (1.5%)

Among caregivers who sought delayed medical care (*n* = 24), the most commonly reported reason for delay was the belief that the illness would resolve spontaneously (*n* = 20). The primary reason for subsequently seeking medical care was a lack of improvement or worsening of the child’s condition.

Among 166 caregivers who did not seek medical care, 124 (74.7%) cited that they perceived that the illness was mild, followed by prior experience with similar symptoms reported by 34 (20.5%) caregivers. Less commonly reported reasons included the belief that traditional medicine is more effective 6 (3.6%) and the perception that the illness did not require medical treatment 6 (3.6%). Transportation difficulties 4 (2.4%) and high medical costs 2 (1.2%) were infrequently reported. Other reasons included the belief that the illness strengthens the child’s immunity, the child’s intolerance to medication, the presence of a family member on the medical staff, and the parents’ busy schedules. All of these were mentioned by a limited number of caregivers and therefore were not included as primary causes.

### Health services characteristics

In terms of service characteristics, most caregivers reported that medical services were accessible, acceptable, and effective. Opinions regarding the cost of services varied. Detailed percentages are presented in Table 3.

**Table 3:** Caregivers’ opinions about health services (*n* = 427)

Item		Frequency (*n*% )
Is it easy for you to reach health services?	Yes	351 (82.2%)
	Sometimes	46(10.8%)
	No	30 (7.0%)
Do you accept to treat your child in hospitals, clinics, and primary healthcare centers?	Yes	372 (87.1%)
	Sometimes	38 (8.9%)
	No	17 (4.0%)
Is the cost of medical care affordable for you?	Yes	254 (59.5%)
	Sometimes	122 (28.6%)
	No	51 (11.9%)
Are the medical services good?	Yes	295 (69.1%)
	Sometimes	105 (24.6%)
	No	27 (6.3%)

Caregivers’ perspectives on modern and traditional medicine showed contrasting patterns. A total of 343 (80.3%) fully trusted the efficacy and safety of modern medicine, while 70 caregivers (16.4%) responded that it is sometimes safe and effective. Only 14 caregivers (3.3%) don’t trust the safety and efficacy of modern medicine at all. On the other hand, opinions on traditional medicine were more varied: only 119 caregivers (27.9%) stated that it is safe and effective, 177 caregivers (41.5%) indicated that traditional medicine is sometimes safe and effective, and 131 (30.7%) perceived it as unsafe or ineffective.

### Relationship of healthcare-seeking behaviors with caregivers, children, illness, and service characteristics

Table 4 shows the relationship between HSB and caregivers and illness characteristics, which was determined by the chi-square test for independence and multivariate logistic regression (95% CI). According to the chi-square test, there was a statistically significant difference in the proportion of appropriate HSB across caregivers’ educational level (*P* = 0.022) and place of residence (*P* = 0.027). Caregivers of children with fever, diarrhea, difficulty breathing, and severe illness had a higher proportion of appropriate HSB compared to their counterparts (p-values: 0.002, 0.036, 0.005, and < 0.001, respectively). Regarding the caregivers’ marital status, it was impossible to find a correlation, as only one caregiver was single, and all of the others were married.

**Table 4:** Relationship between healthcare seeking behaviors and caregivers’, children’s, and illness characteristics

	Appropriate HSB		Chi-square (*P*-value)	Logistic regression		
	*n*	%		*P*-value	Odds ratio	95% C.I.
Caregivers characteristics						
Caregiver’s age (years)						
≤ 29	121	55.0%	0.047 (0.829)			
> 29	116	56.0%				
Occupation						
Employed	61	53.9%	0.144 (0.704)			
Unemployed	176	53.2%				
Educational level						
Secondary school or higher	215	54.0%	5.221 (0.022)^a^	0.203	0.55	0.22–1.38
Primary school or less	22	75.9%				
Place of residence						
City	155	52.0%	4.865 (0.027)^a^	0.014^a^	0.56	0.36–0.89
Village or camp	82	63.6%				
Children characteristics						
Child age (months)						
≤ 12	91	59.5%	1.524 (0.217)			
> 12	146	53.3%				
Child sex						
Male	127	54.3%	0.317 (0.573)			
Female	110	57.0%				
Birth order						
The first or second	135	51.9%	3.451 (0.063)			
After second	102	61.1%				
Number of siblings						
≤ 2	168	54.0%	1.021 (0.312)			
> 2	69	59.5%				
Illness characteristics						
Fever						
Yes	177	60.6%	9.776 (0.002)^a^	0.042^a^	1.61	1.02–2.54
No	60	44.4%				
Cough						
Yes	108	57.1%	0.369 (0.544)			
No	129	54.2%				
Diarrhea						
Yes	73	48.7%	4.376 (0.036)^a^	0.064	0.66	0.43–1.02
No	164	59.2%				
Difficulty of breathing						
Yes	63	68.5%	7.993 (0.005)^a^	0.019^a^	1.87	1.11–3.16
No	174	51.9%				
Perception of illness						
Severe	147	67.4%	25.657(0.000)^a^	0.000^a^	2.43	1.60–3.72
Not severe	90	43.1%				

^a^*P*-value < 0.05

After adjusting the independent variables in a well-fitting model using multivariate logistic regression analysis, caregiver education level and diarrhea no longer had a significant relationship with HSB. According to multivariate logistic regression analysis, caregivers who live in cities are two times less likely to engage in appropriate HSB than those who live in villages or camps (OR = 0.57, 95% CI: 0.36–0.90). Caregivers of children with fever or difficulty breathing were 1.6 and 1.9 times more likely to engage in appropriate HSB (OR = 1.61, 95% CI: 1.02–2.54) and (OR = 1.90, 95% CI: 1.11–3.16), respectively. Perceiving illnesses as severe was 2.4 times more likely to seek appropriate HSB as compared to not severe (OR = 2.43, 95% CI: 1.60–3.72).

The analysis of children’s characteristics and health service characteristics showed no statistically significant differences with HSB. The P-values for all children’s characteristics and service characteristics, calculated using the Chi-square test, exceeded 0.05. While opinions varied regarding the effectiveness and safety of modern and traditional medicine, the analysis found no statistically significant relationship between these views and HSB.

## Discussion

Our findings revealed that over half of caregivers (55.5%) exhibited appropriate HSB by seeking medical care from suitable health facilities within the first three days of their child’s illness, which is relatively comparable to findings in a study conducted in Yemen (51.4%), higher than studies conducted in rural Nigeria (25.3%), and rural western Kenya (30.4%) this may be partly attributed to contextual differences, including greater religious and sociodemographic diversity in the Nigerian population, which has been shown to influence care-seeking behaviors in complex ways [15, 16, 22], . While the overall pattern of care-seeking aligns with existing global literature, the present study demonstrates how such patterns are operationalized within a conflict-affected setting, where movement restrictions, political instability, and fragmented health services delay timely access to care. In contrast, the prevalence of appropriate HSB in our study is lower than the 82.1% reported in a study conducted in Dangila, North West Ethiopia [23]. Lower appropriate healthcare-seeking behavior in Palestine may be explained by movement restrictions, political instability, and fragmented health services, which delay timely access to care. In contrast, Ethiopia’s community-based health extension program promotes early care-seeking through local outreach. This gap provides a window to learn from Ethiopia’s model to strengthen community engagement and accessibility in Palestine [24].

In our study, 5.6% of the caregivers sought medical care after more than 3 days of the onset of symptoms, with the main reason for the delay being the thought that the illness would resolve. Similarly, another study reported the same reason for the delay [15]. This delay has many consequences, including increased risk of complications and reduced treatment efficacy. Educating the caregivers about the sequelae of delaying medical attention and about the importance of timely healthcare access could help reduce these avoidable delays [25].

Among those who didn’t seek medical care at all, several factors were cited by the caregivers to have contributed to their decisions not to seek medical care at all, reflecting a combination of perceptual, experiential, cultural, and contextual barriers that influence health-seeking behavior. First, the most common reason stated by (74.7%) of the care givers that the illness appeared mild, which is consistent with findings from Nepal, Ethiopia, Nigeria, and Yemen [15, 23, 26, 27]. This reflects a widely reported phenomenon in which caregivers judge the severity of illness as the main determinant for seeking care. Research indicates that when symptoms are perceived as minor or self-limiting, caregivers are significantly less likely to pursue professional medical attention, often relying on home remedies or waiting for the illness to resolve spontaneously [28]. Another barrier to seeking appropriate healthcare was caregivers’ confidence in managing recurrent illnesses themselves. In this study, 20.5% of caregivers reported not seeking care because of prior experience with the same symptoms. However, this approach assumes that each episode will behave the same as previous ones and may fail to account for important changes, such as the child’s age, worsening or different symptom severity, or the emergence of new or complicating health conditions. As a result, there is a need for public education on assessing illness severity and recognizing when medical intervention is required, as relying solely on experience may not always produce safe outcomes. It is also important for healthcare providers to explain to caregivers that each episode of illness should be evaluated based on current factors when encountering recurring symptoms in children.

To a lesser extent, 3.6% of caregivers in this study believed the illness did not require medical attention, so they didn’t seek medical care, compared with 31% in Yemen. This difference may be a consequence of differences in educational attainment between the study populations: in our study, 68% of caregivers had higher education, whereas in the Yemeni survey, around 50% had only primary education, and 27% had no formal education. Higher education in our population may increase awareness of potential risks and the importance of professional care, whereas lower education in the Yemeni sample may have contributed to a greater reliance on personal judgment and a perception that medical care was unnecessary [15]. Another 3.6% of caregivers reported that they did not seek medical care because they believed traditional medicine was a better alternative, indicating exclusive reliance on traditional practices. This proportion is relatively small and does not contradict existing evidence from Palestine showing widespread use of complementary and alternative medicine (CAM), as 63% of caregivers in our study reported using self-treatment before seeking medical care, which often included CAM practices such as herbal remedies, cold applications, and common home treatments mentioned during open-ended interviews. These findings are consistent with previous Palestinian research demonstrating that CAM is frequently used alongside medical treatment, before seeking professional care, or as a complementary, not necessarily exclusive, approach, driven by perceptions that CAM is more natural, safer, and less invasive than conventional medicine [14]. Finally, 1.2% of caregivers stated high medical costs as a reason for not seeking care. This finding is similar to a study in northwest Ethiopia [23], where cost was also reported as one of the least common reasons for avoiding medical care. In contrast, high costs were a significant factor in a Nigerian study, with 41.4% citing them as a reason for not seeking treatment [29]. The lower concerns in our study may be attributed to the availability of various health insurance options in Palestine, which are supported by the government, private, and international organizations, including UNRWA [30, 31].

A notable aspect of our study was the prominent role mothers played in healthcare decisions, with 94.6% of respondents being mothers and 76.1% making the decision alone. This finding reflects prevailing cultural norms in many societies, including Palestine, where women are traditionally assigned primary responsibility for family care, particularly during periods of illness. Within Palestinian culture, caregiving is commonly perceived as a woman’s role, encompassing care for children, older adults, and other ill family members, whereas males are usually assigned to gain income [32, 33]. Another possible explanation for this finding is that in our country, 71% of the working population is male, while females account for only 17% [34], so fathers may have been working during the time of data collection, limiting their involvement. In light of these findings, interventions aimed at improving healthcare-seeking behaviors should prioritize educating mothers, who are frequently the primary decision-makers in child healthcare.

In terms of caregiver demographics, we found no notable differences in appropriate HSB across caregivers according to their age, occupation, or education level. Although differences were observed by place of residence. Caregivers residing in villages and camps were twice as likely as city residents to seek appropriate care, which may reflect the availability of primary healthcare services in rural areas, including primary healthcare centers and 24-hour emergency services in nearly every village, as well as UNRWA primary healthcare centers in each camp [6]. This contrasts with findings in Ethiopia, where urban residency was linked to increased appropriate care-seeking behavior, implying that localized factors influence health access differently across regions [35].

Assessing child characteristics, our study found no clear differences in caregivers’ HSB across them. This suggests that caregivers’ decisions to seek medical care are less influenced by the child’s demographic profile and may be more strongly guided by other considerations. Other studies have shown varied outcomes in this area. Some studies reported that younger children, particularly infants, elicit a faster caregiver response due to perceived vulnerability [23], whereas others, such as ours, show that caregivers may use similar care-seeking strategies regardless of the child’s age or other demographic factors [15]. This finding is interesting as it suggests that caregivers in our setting view HSB as equally necessary for all children and, as a result, health initiatives promoting prompt HSB could be applied broadly, without needing customization for different child profiles.

In terms of illness characteristics, caregivers in our study were more likely to seek appropriate medical care when their child experienced breathing difficulty. This is consistent with findings from other studies, which showed that respiratory symptoms frequently prompt caregivers to seek urgent medical attention [15, 36, 37]. This could be because respiratory symptoms may be perceived as distressing by mothers [38]. Fever was also a significant trigger for care-seeking, with children presenting with fever being 1.6 times more likely to be taken for medical evaluation compared with those presenting with other complaints. Although our interviews did not explore the underlying reasons why fever appeared more concerning to caregivers, a previous study among the same population of Palestinian caregivers found that parents universally believed fever could lead to harmful outcomes, such as brain damage or dehydration, if not properly managed [39].

The study also highlighted that caregivers were more likely to seek medical care for illnesses perceived as severe. This finding is in line with many studies [16, 18, 26]. Caregivers in our study who perceived their child’s illness as severe were 2.5 times more likely to seek appropriate medical care because when caregivers perceive the illness as severe, they seek medical attention out of fear of complications. These results highlight the importance of educating caregivers on early recognition of severe symptoms as well as the need for healthcare providers to offer proper counseling to caregivers. Providers can empower caregivers to make better decisions in the future by educating them on the warning signs that require medical attention.

Regarding health service factors, we found no statistically significant relationship between HSB and service accessibility, affordability, or quality perceptions. This stands in contrast to previous researches which highlights distance, facility quality, and cost as significant factors influencing HSB [40–42]. Our interpretation is that, in this setting, structural barriers are relatively uniform across the population, limiting their discriminatory effect on healthcare-seeking behavior. We assume that when access constraints are shared broadly, individual decisions may be driven less by facility-related factors and more by caregivers’ beliefs, illness interpretations, and prior experiences with childhood illness.

Finally, caregivers demonstrated high confidence in modern medicine (80.3% rated it as safe, while their views on traditional medicine were more mixed, with only 27.9% considering it completely safe and effective, and 41.5% seeing it as sometimes effective. This mixed perception may stem from individual experiences and community beliefs about traditional remedies. Given these attitudes, there is a need to educate caregivers about evidence-based practices and support informed decisions regarding the use of traditional medicine.

The study’s main strength is that it focuses on identifying the prevalence of appropriate HSB and the factors that influence it, which has not been addressed in previous studies in Palestine. Furthermore, this study has numerous methodological strengths, including the fact that the interview questionnaire is superior to the self-administered questionnaire and that the sample is representative of various cultural backgrounds. In addition, although convenience sampling was applied, participants were recruited from both urban and rural primary healthcare centers across four governorates, contributing to cultural and socioeconomic diversity within the study. The study faced several limitations that may impact the generalizability of the findings. First, Although the study was framed as an analytical cross-sectional design, the sample size was calculated to estimate the prevalence of HSB rather than to ensure sufficient statistical power to detect associations between HSB and potential determinants; therefore, some associations may have been underpowered, additionally the sample size included only a small number of residents from refugee camps, as UNRWA declined to participate in the study. Additionally, ongoing political challenges in the Palestinian territories made it difficult to access these populations, further limiting the representativeness of the sample for the entire country. Moreover, we relied on a prestructured interviewer-administered questionnaire, which, while facilitating standardized data collection, may not have captured the full range of factors influencing health-seeking behavior (HSB). Third, caregivers’ responses could not be independently verified, introducing the possibility of recall bias. Finally, several factors potentially relevant to HSB, such as health literacy, social support, or previous healthcare experiences, were not measured, raising the possibility of unmeasured confounding. Given the complexity of HSB and the influence of cultural and contextual factors, we believe this topic would be better explored through qualitative research, which could provide deeper insights into caregivers’ perceptions, motivations, and decision-making processes.

## Conclusion and recommendations

This study provides context-specific evidence on how caregivers’ health-seeking behaviors are shaped within a conflict-affected area with a fragmented health care system. The study’s findings indicate that more than half of caregivers sought healthcare in a timely and appropriate manner. However, many initially relied on traditional or self-treatment methods, which caused delays in professional care. Caregivers’ perceptions of illness severity, specific symptoms (particularly respiratory difficulties), and rural residency all had a significant impact on timely healthcare seeking. In contrast, characteristics of health services and children had no significant impact on caregivers’ HSB, implying that caregivers’ decisions may be more strongly influenced by cultural beliefs and perceptions of illness.

There is also a clear need for public health programs to educate caregivers on the potential risks and appropriate role of traditional practices, given their widespread use. Emphasizing early intervention for severe symptoms is essential to ensure timely access to healthcare. In addition, future studies should adopt qualitative approaches to gain deeper insights into caregivers’ perspectives and motivations, particularly regarding the use of traditional medicine. Understanding its types, prevalence, and cultural drivers can inform health education strategies and help service providers deliver culturally sensitive care.

## Data Availability

All datasets generated and analyzed during the current study are available from the corresponding authors upon reasonable request.
